# The treatment of resistant staphylococcal infections

**DOI:** 10.12688/f1000research.17718.1

**Published:** 2020-02-26

**Authors:** Joseph John Jr

**Affiliations:** 1 Lowcountry Infectious Diseases, Charleston, South Carolina, USA; 2 Division of Infectious Diseases, Medical University of South Carolina, Rutledge Tower, Charleston, South Carolina, USA

**Keywords:** staphylococcal infections, antimicrobial resistance

## Abstract

*Staphylococcus aureus *of the many staphylococcal species is the most common cause of both skin and soft tissue infection and severe staphylococcal infections including *Staphylococcus aureus* bacteremia (SAB). Many antibiotics are active against the staphylococci, yet over the last 40 years antibiotic resistance, particularly resistance to beta-lactam antibiotics, has plagued antimicrobial therapy. The term “methicillin resistance” is a historic term and now refers to the ability of staphylococci, in particular methicillin-resistant *Staphylococcus aureus *(MRSA), to resist the action of beta-lactam antibiotics. This resistance is encoded by the *mec*A gene carried in a complex genetic cassette, SCC *mec*. **Vancomycin and old antibiotics remain the keystone of treatment for resistant staphylococci. Other newer agents, and some older agents, show good activity against resistant staphylococci which are the focus of this review: trimethoprim-sulfamethoxazole, ceftaroline, daptomycin, fosfomycin, linezolid, dalbavancin, televancin, and omadacycline. Other agents with novel mechanisms of action are under development, for use as single anti-staphylococcal agents or for combination use to augment the action of the primary anti-staphylococcal agent. Vancomycin therapy carries specific risks, particularly renal dysfunction, but despite its foibles, vancomycin remains the standard of care for the treatment of resistant staphylococcal infections. Some clinicians implement an early switch from vancomycin at the earliest signs of renal dysfunction. The near horizon holds promise also of augmentation of both cellular and humoral responses to staphylococcal infection. Pending newer clinical trials that show clear superiority of one anti-staphylococcal agent over another or over vancomycin, it will remain to expert clinical judgment in determining antibiotic choice and duration of anti-staphylococcal therapy.

## Background 

*Staphylococcus aureus*, one of many species of staphylococci, usually causes skin and soft tissue infections (SSTIs) in humans. Infections caused by antibiotic-resistant strains of *S. aureus*—one of the “ESKAPE” pathogens ( *Enterococcus faecium*, *S. aureus*, *Klebsiella pneumoniae*, *Acinetobacter baumannii*, *Pseudomonas aeruginosa*, and *Enerobactger species*)—have become increasingly difficult to treat. This emergent antimicrobial resistance has prompted development, marketing, and use of newer anti-staphylococcal antimicrobials. Non-cutaneous and non-soft tissue infections, though severe, are less frequent than SSTIs. Both SSTIs and non-SSTIs may be associated with *S. aureus* bacteremia (SAB). SAB as a separate entity retains a singular notoriety because of its propensity to produce metastatic abscesses and occult extra-vascular infection ^1^. When SAB occurs without an identifiable origin, clinicians must always rule out *S. aureus* endocarditis which requires prolonged intravenous (IV) antibiotic therapy. Still SSTI and (to a much lesser degree) SAB remain the major focus for clinical trials in hopes of bringing new anti-staphylococcal antimicrobials to the marketplace. Clinical trials of new antimicrobials have relied on study designs to show the study agent to be only non-inferior (not superior) to standard therapy.

Susceptibilities of *S. aureus* infections are traditionally divided between methicillin-susceptible (MSSA) and methicillin-resistant (MRSA), although methicillin resistance is a historic term since it was replaced years ago by the alternative semi-synthetic penicillins like oxacillin, nafcillin, and cloxacillin. Over the past four decades, the antimicrobial therapy of resistant staphylococci has moved through multiple iterations. When MRSA first appeared, those early strains contained a complex SCC *mec* genetic cassette containing multiple antimicrobial resistance genes ^2^. Gradually, SCC *mec* has evolved into many subtypes by adding and deleting multiple resistance genes. Nosocomial clones of MRSA (USA 100 and USA 200) in the early 1970s contained a complex SCC *mec* which evolved paradoxically in the 1990s to a much less complex and less resistant SCC *mec* in community-associated USA 300 clones. USA 300 clones have caused a worldwide pandemic and continue to infect many patients not only in the community but also within hospitals.

Vancomycin was one of the few bactericidal antimicrobial agents to which early MRSA strains were susceptible. Today, vancomycin remains the mainstay of initial therapy for resistant staphylococcal infections. Over 60 years, however, have passed since its introduction, its widespread use and a “creeping” increase in minimum inhibitory concentrations (MICs), prompting the pharmaceutical industry to develop new anti-staphylococcal antimicrobials. Multiple new anti-staphylococcal agents have entered the marketplace, lowering the threshold for moving beyond vancomycin pre-eminence to a new age of anti-staphylococcal antimicrobial chemotherapy ^3^.

In this review, we will highlight the newest evidence of how well the new agents work to reduce morbidity and mortality due to staphylococcal infections, particularly in comparison with the established efficacy of vancomycin. A current PubMed search, Treatment for Staphylococcal Infections, yields nearly 11,000 entries. We aimed to concentrate on some “bottom line” recommendations for newer therapies as alternatives to vancomycin. Additionally, for the most part, we will be addressing the antimicrobial therapy of pathogenic coagulase-positive staphylococci, *S. aureus*, even though infections due to multiple species of coagulase-negative staphylococci (CNS) constitute a real and present danger for producing human infection ^4^. Most, but not all, of the recommendations in our review will apply as well to CNS, which in general exhibit broader antimicrobial resistance than their coagulase-positive counterparts.

## Alternatives to vancomycin therapy

Table 1 lists antimicrobials that are now available as alternatives to vancomycin and that we will consider in this review. Even with multiple alternatives, vancomycin remains a mainstay of anti-staphylococcal therapy. Indeed, despite its potential for producing renal toxicity and (to a much lesser extent) ototoxicity, vancomycin remains the first choice in community hospitals for suspected serious SSTI and invasive disease due to staphylococci. In this era of formatting clinical trials to show non-inferiority, we know, *a priori*, that our review will be challenged to find any superiority of newer anti-staphylococcal over vancomycin.

**Table 1.** Alternatives to semi-synthetic penicillins and cephalosporins for the treatment of resistant staphylococcal infections.

• Ceftaroline • Daptomycin • Fosfomycin • Linezolid • Oritavancin/Dalbavancin • Telavancin • Omadacycline

Whether vancomycin or an alternative is chosen as the therapy of choice, out-patient parenteral antibiotic therapy (OPAT) can reduce the in-patient costs substantially. One study found that oritavancin used in OPAT resulted in savings of $1752 to $6475 per patient ^5^.

## Trimethoprim sulfamethoxazole

To put the therapeutic issues into perspective, let us start with consideration of the newest of trials involving one of the oldest anti-staphylococcal agents, trimethoprim/sulfamethoxazole (TMP-SMZ). Just how effective is TMP-SMZ in treating staphylococcal infections? In one major trial, TMP-SMZ or placebo was used for a 7-day course following drainage of a staphylococcal abscess ^6^. Test of cure was carried out at 14 to 21 days. Failure was continued fever with relative amounts of erythema or swelling at day 3 or 4, days 8 to 10, or days 14 to 21. The primary test resolution of the abscess occurred at 7 to 14 days. If patients failed, they discontinued the treatment or placebo and could undergo further drainage. In all, 1265 patients enrolled and 1247 were assigned treatment. For placebo and treatment, respectively, 607 and 606 were included in modified intention to treat. For those groups, 509 and 504, respectively, completed an extended follow-up visit. The cure rates were 80.5% in the TMP-SMZ group and 73.6% in the placebo drainage-alone group. In the per-protocol group, clinical cure rates were 487 (92.9%) out of 524 in the treatment group and 457 (85.7%) out of 533 in the placebo group. Differences in these two groups were highly significant. Adverse events were similar in the two groups. There were no cases of *Clostridium difficile*–associated diarrhea. In TMP-SMZ–susceptible strains of *S. aureus*, TMP-SMZ clearly remains a reasonable alternative to beta-lactam therapy ^6^.

This study provides a perspective for other more recent skin and soft tissue studies that we will discuss. Although it is important to state that adjunctive antibiotics in this study of skin abscesses were associated with small but statistically significant improved outcomes, it is also important to note that three quarters of the abscesses did improve without antibiotics. Even large studies like this one have drawbacks. Not all of these abscesses had a proven isolate of *S. aureus*. Only about 63% in each group had an MRSA or MSSA isolated. Thus, if there is a skin abscess to drain, drainage plus an antimicrobial will probably produce cure in over 90% of patients.

Although vancomycin was not used as a comparator in this study, it is likely that results would have been comparable.

Of the studies we will consider, many will not involve drainable SSTIs or the intent of the study was not to initiate drainage. Like TMP-SMZ, many older antimicrobial agents (including ciprofloxacin, clinidamycin, doxycycline, and rifampicin) remain active against resistant staphylococci, particularly of community origin. Consideration of these old soldiers still relevant to the treatment war against staphylococci is beyond the scope of this review.

As mentioned above, Table 1 lists newer antimicrobials which are now available as alternatives to vancomycin or TMP-SMZ, which we will consider below in this brief review.

## Ceftaroline

Ceftaroline is a cephalosporin with activity against MRSA. For years, clinicians have been trained to avoid cephalosporins for the treatment of MRSA. Finally, this particular cephalosporin that could bind to PBS2A and bypass the block to cephalosporin efficacy in MRSA strains became available over the last decade. Starting with the CANVAS 1 and CANVAS 2 studies, it was clear that ceftaroline was at least as effective as vancomycin in the treatment of SSTI ^7^. Day 3 response rates were 74.0% (296/400) for ceftaroline and 66.2% (263/397) for vancomycin. Community hospitals around the country have been slow to place ceftaroline on the general formulary or have restricted its use to infectious disease consultants. A growing experience suggests that ceftaroline will become a mainstay of anti-staphylococcal therapy for methicillin-resistant staphylococci.

## Daptomycin

Daptomycin is a cyclic lipopeptide that has become a frequent alternative to vancomycin, except in the case of staphylococcal pneumonia since the compound is highly bound to pulmonary surfactant. A landmark study of SAB showed that daptomycin was non-inferior to vancomycin in the outcomes of SAB; however, response rates were low for both compounds ^8^. Nevertheless, daptomycin is now supplanted for vancomycin as soon as problems such as clinical non-response or impending renal toxicity arise. One very specific though technical concern prompting more daptomycin use has been the increasing MIC equaling or exceeding 2 μg/mL of vancomycin for staphylococci. One classic case-controlled study suggested improved outcomes for daptomycin over vancomycin for bloodstream infections due to MRSA. Some clinicians, however, advise increasing the dosage above 6 mg/kg per day for daptomycin in SAB and severe invasive infections. In the algorithm arm, a very recent trial employing an algorithm-based therapy versus usual care urged the use of vancomycin in the treatment of MRSA ^4^. Daptomycin was the only alternative to vancomycin in the algorithm arm. A major finding in that elegant study was the superiority of the algorithm arm in the MRSA group. Controlled trials for the treatment of staphylococcal osteomyelitis using daptomycin versus comparators are in process. Overt muscle toxicity due to daptomycin, an initial clinical concern, has been rare, although some elevation of muscle enzymes is seen in a predictable percentage of cases.

## Fosfomycin

This powered agent, which is becoming more popular in the US, is used for short-term therapy of urinary tract infections. It has broad-spectrum antibacterial activity, including excellent activity against the staphylococci. It is well concentrated in urine but not in tissue. It can be given in combination with other anti-staphylococcal agents ^9^. At present, it should not be used as monotherapy for severe staphylococcal infections of the deep tissues.

## Linezolid

Sold under the brand name Zyvox (Pfizer Inc., New York, NY, USA), linezolid, which has been in use for almost 20 years, was hampered during its early usage by its high cost. It is still relatively expensive but has become a valued alternative to vancomycin in moderately severe staphylococcal infection, particularly for lung infections with MRSA and for SSTI. A 5-year (2011–2015) look back at the susceptibility of 3031 isolates of *S. aureus* to linezolid showed that more than 99.9% retained susceptibility ^10^. Linezolid has become a mainstay alternate to vancomycin for severe non-endothelial staphylococcal infections. Additionally, there are observations that vancomycin monotherapy may be sorely insufficient to treat MRSA in children with concomitant influenza ^11^.

Tedisolid was approved in 2014 for parenteral and oral use at a dosage of 200 mg per day. Like linezolid, it produces occasional severe adverse effects, including thrombocytopenia, neuropathy, and even optic neuropathy.

## Oritavancin/Dalbavancin

Dalbavancin is a lipoglycopeptide with an extremely long half-life. It can be given weekly for the treatment of staphylococcal infections. Expense is a major consideration and some insurance companies in the US will not cover the cost. Nevertheless, both dalbavancin and its cousin, oritavancin, have very low MICs for the staphylococci, including hetroresistant *S. aureus*. Use of dalbavancin for endocarditis suggests a potential role ^12^, but the number of treated patients with endocarditis is small. One patient with tricuspid valve MRSA endocarditis failed 4 weeks of dalbavancin therapy ^13^.

Oritavancin also has an extremely long half-life and is marketed as a single-dose agent of 1200 mg (given as an IV infusion slowly over the course of 3 hours), which in clinical trials (SOLO I and SOLO II) has been shown to be non-inferior to vancomycin ^5^. Patients should be monitored for hypersensitivity during IV infusion. The complexity of its use and risk of hepatic side effects will hang over widespread use of oritavancin.

## Televancin

Televancin, approved by the US Food and Drug Administration almost a decade ago, is another lipoglycopeptide derived from vancomycin and is highly active against staphylococci. For the therapy of SSTI, the clinical success rate approaches 90% but televancin is available only as an IV infusion ^14^. Nephrotoxicity has prompted a black box warning for increased mortality in patients with moderate-to-severe kidney impairment. Warnings include the risk of prolonged QT interval, hypersensitivity reactions, and prolongation of prothrombin time and activated partial thromboplastin time, the latter of which is a laboratory artifact not having an effect on coagulation.

## Tigecycline

Tigecycline, initially marketed as a broad-spectrum therapy for intra-abdominal abscesses, retains good inherent activity against staphylococci. It has yet to find its place as a primary monotherapy for staphylococcal infections. Yet its excellent activity against MRSA and its penetration into bone and biofilm suggest that it may come to be useful as monotherapy or in combination therapy for infected wounds, diabetic foot infections, and osteomyelitis but not pneumonias ^15^.

Tigecycline has been overshadowed by the introduction of a new once-daily parenteral as well as an oral preparation of omadacycline (NUZYRA). Omadacycline was designed to overcome tetracycline resistance and is approved for community-acquired pneumonia and SSTI ^16^.

## Future directions

Imperative to treating antimicrobial resistant bacteria, including the staphylococci, is preservation of current agents and development of new agents. The Infectious Diseases Society of America calls for the development of 10 new agents by 2020, a tall order indeed given that costs to market a new antimicrobial agent are hundreds of millions of dollars. Many new agents with novel mechanisms of antibacterial and anti-staphylococcal activity are in development. In this brief review, we have only touched on current marketed alternates to older therapies ^17^. For an example of future novel agents, certain endolysins isolated from bacteriophages when delivered through novel delivery systems quickly kill *S. aureus* ^18^.

For all its foibles, vancomycin remains the standard of care for therapy of resistant staphylococci, although a recent survey of adult infectious disease physicians from five large medical centers shows an inclination to change to vancomycin alternatives ^19^. Many patients, perhaps millions, with severe *S. aureus* infections have been cured with vancomycin therapy. Studies of newer agents that show merely non-inferiority to vancomycin may not be sufficiently designed to show superior rates of cure which would prompt a change of allegiance from vancomycin. One multicenter study looked at the failure of standard therapy to sterilize blood cultures in MRSA bloodstream infections ^20^. When ceftaroline was used as salvage therapy in 211 bacteremic patients, cure rates approached 70% ^21^. Treatment algorithms also can assist early choice of therapy for staphylococcal infections ^21^. Newer systematic algorithms to guide testing and treatment to guide clinicians in a sequential approach to staphylococcal bacteremia were recently published ^3^. Use of the algorithm resulted in a non-inferior rate of clinical success, whereas, in uncomplicated bacteremia, the use of the algorithm reduced the mean duration of therapy from 6.2 to 4.4 days.

Combination therapy for severe staphylococcal infections offers a new horizon for study. To date, there are several studies to note that report good results, one using daptomycin plus ceftaroline ^22^, one using vancomycin plus cefazolin ^23^, and one employing daptomycin plus beta-lactam combinations ^24^. Surely, other combinations using newer agents will be employed over the next decade addressing the question of whether we can improve outcomes over monotherapy.

Enhancement of effective host responses is a growing trend to assistance clearance or prevent staphylococcal infections. Staphylococcal vaccines have been notoriously ineffective. Because cellular immunity plays a pivotal role in host resistance to staphylococci, the staphylococcal research field has newly focused on augmenting cellular host responses. One recent study of so-called immune checkpoint therapy showed that reduction in one CD28 receptor, inducible co-stimulator (ICOS), improves survival in murine staphylococcal pneumonia, probably through the limitation of exaggerated cytokine expression ^25^. Other perturbations of both the cellular and humoral response are likely to find their way into prevention and reduction of morbidity in resistant staphylococcal infections ^26^.

## Conclusions

The therapy of severe staphylococcal infections, including those which produce SAB, remains in flux. It is reassuring that for therapy of these infections there are now alternatives to vancomycin whose efficacy is backed by decent clinical trials. Yet the limitations of small sample size and lack of investment in these clinical trials emphasize the need for expert clinical judgment in determining antibiotic choice and duration of anti-staphylococcal therapy ^27^.

## References

[1] John JF: Staphylococcal infections: emerging clinical syndromes. In D A’Aldeen, K Hiramatsu. *Staphylococcus aureus: Molecular and Clinical Aspects.*Harwood Publishing Limited. West Sussex.2004;1–30. 10.1016/B978-1-898563-96-9.50006-3

[2] Ito T Hiramatsu K Tomasz A: Guidelines for reporting novel mecA gene homologues. *Antimicrob Agents Chemother.* 2012;56(10):4997–9. 10.1128/AAC.01199-12 22869575 PMC3457410

[3] Cunha BA: Vancomycin revisited: a reappraisal of clinical use. *Crit Care Clin.* 2008;24(2):393–420, x-xi. 10.1016/j.ccc.2007.12.012 18361953

[4] Holland TL Raad I Boucher HW: Effect of Algorithm-Based Therapy vs Usual Care on Clinical Success and Serious Adverse Events in Patients with Staphylococcal Bacteremia: A Randomized Clinical Trial. *JAMA.* 2018;320(12):1249–58. 10.1001/jama.2018.13155 30264119 PMC6233609

[5] Deck DH Jordan JM Holland TL: Single-Dose Oritavancin Treatment of Acute Bacterial Skin and Skin Structure Infections: SOLO Trial Efficacy by Eron Severity and Management Setting. *Infect Dis Ther.* 2016;5(3):353–61. 10.1007/s40121-016-0119-9 27370913 PMC5019975

[6] Cocchi P Taccetti G Galli L: Management of skin abscesses. *N Engl J Med.* 2014;370(23):2244–5. 10.1056/NEJMc1404437 24897094

[7] File TM Jr Wilcox MH Stein GE: Summary of ceftaroline fosamil clinical trial studies and clinical safety. *Clin Infect Dis.* 2012;55(Suppl 3):S173–80. 10.1093/cid/cis559 22903949

[8] Fowler VG Jr Boucher HW Corey GR: Daptomycin versus standard therapy for bacteremia and endocarditis caused by Staphylococcus aureus. *N Engl J Med.* 2006;355(7):653–65. 10.1056/NEJMoa053783 16914701

[9] Jia Y: The progress in study of fosfomycin. *Infection International.* 2018;6(3):88–92. 10.1515/ii-2017-0162

[10] Pfaller MA Mendes RE Streit JM: Five-Year Summary of *In Vitro* Activity and Resistance Mechanisms of Linezolid against Clinically Important Gram-Positive Cocci in the United States from the LEADER Surveillance Program (2011 to 2015). *Antimicrob Agents Chemother.* 2017;61(7): pii: e00609-17. 10.1128/AAC.00609-17 28483950 PMC5487612

[11] Randolph AG Xu R Novak T: Vancomycin Monotherapy May Be Insufficient to Treat Methicillin-resistant *Staphylococcus aureus* Coinfection in Children With Influenza-related Critical Illness. *Clin Infect Dis.* 2019;68(3):365–72. 10.1093/cid/ciy495 29893805 PMC6336914

[12] Tobudic S Forstner C Burgmann H: Dalbavancin as Primary and Sequential Treatment for Gram-Positive Infective Endocarditis: 2-Year Experience at the General Hospital of Vienna. *Clin Infect Dis.* 2018;67(5):795–8. 10.1093/cid/ciy279 29659732

[13] Steele JM Seabury RW Hale CM: Unsuccessful treatment of methicillin-resistant *Staphylococcus aureus* endocarditis with dalbavancin. *J Clin Pharm Ther.* 2018;43(1):101–3. 10.1111/jcpt.12580 28628223

[14] Smith WJ Drew RH: Telavancin: a new lipoglycopeptide for gram-positive infections. *Drugs Today (Barc).* 2009;45(3):159–73. 10.1358/dot.2009.45.3.1343792 19436839

[15] Griffin AT Harting JA Christensen DM: Tigecycline in the management of osteomyelitis: a case series from the bone and joint infection (BAJIO) database. *Diagn Microbiol Infect Dis.* 2013;77(3):273–7. 10.1016/j.diagmicrobio.2013.07.014 24029432

[16] Chambers HF: Omadacycline - The Newest Tetracycline. *N Engl J Med.* 2019;380(6):588–9. 10.1056/NEJMe1900188 30726683

[17] Bassetti M Russo A Carnelutti A: Antimicrobial resistance and treatment: an unmet clinical safety need. *Expert Opin Drug Saf.* 2018;17(7):669–80. 10.1080/14740338.2018.1488962 29897796

[18] Haddad Kashani H Schmelcher M Sabzalipoor H: Recombinant Endolysins as Potential Therapeutics against Antibiotic-Resistant *Staphylococcus aureus*: Current Status of Research and Novel Delivery Strategies. *Clin Microbiol Rev.* 2018;31(1): pii: e00071-17. 10.1128/CMR.00071-17 29187396 PMC5740972

[19] Banniettis N Beekmann SE Polgreen PM: Management Practices for Methicillin-Resistant *Staphylococcus aureus* Bacteremia by Adult Infectious Diseases Physicians. *Open Forum Infect Dis.* 2018;5(5): ofy093. 10.1093/ofid/ofy093 29876367 PMC5961149

[20] Zasowski EJ Trinh TD Claeys KC: Multicenter Observational Study of Ceftaroline Fosamil for Methicillin-Resistant Staphylococcus aureus Bloodstream Infections. *Antimicrob Agents Chemother.* 2017;61(2): pii: e02015-16. 10.1128/AAC.02015-16 27895012 PMC5278749

[21] Crossley KB John J: Treatment of staphylococcal infections In Crossley and Archer (eds). *Staphylococci in Human Disease*Wiley-Blackwell 2nd Edition.2009;570–593. 10.1002/9781444308464

[22] Geriak M Haddad F Rizvi K: Clinical Data on Daptomycin plus Ceftaroline versus Standard of Care Monotherapy in the Treatment of Methicillin-Resistant *Staphylococcus aureus* Bacteremia. *Antimicrob Agents Chemother.* 2019;63(5): pii: e02483-18. 10.1128/AAC.02483-18 30858203 PMC6496065

[23] Trinh TD Zasowski EJ Lagnf AM: Combination Vancomycin/Cefazolin (VAN/CFZ) for Methicillin-Resistant *Staphylococcus aureus* (MRSA) Bloodstream Infections (BSI). *Open Forum Infect Dis.* 2017;4(Supp 1):S281 10.1093/ofid/ofx163.631

[24] Jorgensen SCJ Zasowski EJ Trinh TD: Daptomycin plus beta-lactam combination therapy for methicillin-resistant *Staphylococcus aureus* bloodstream infections: a retrospective, comparative cohort study. *Clin Infect Dis.* 2019; pii: ciz746. 10.1093/cid/ciz746 31404468

[25] Pires S Jacquet R Parker D: Inducible Costimulator Contributes to Methicillin-Resistant *Staphylococcus aureus* Pneumonia. *J Infect Dis.* 2018;218(4):659–68. 10.1093/infdis/jix664 29378030 PMC6047441

[26] Dupont CD Scully IL Zimnisky RM: Two Vaccines for *Staphylococcus aureus* Induce a B-Cell-Mediated Immune Response. *mSphere.* 2018;3(4): pii: e00217-18. 10.1128/mSphere.00217-18 30135219 PMC6106056

[27] Perencevich EN Malani PN: Treatment Algorithms for Staphylococcal Bacteremia: Improving Clinical Care and Enhancing Antimicrobial Stewardship. *JAMA.* 2018;320(12):1243–4. 10.1001/jama.2018.13315 30264099

